# Investigation of the impact of blur under mobile attentional orientation using a vision simulator

**DOI:** 10.1371/journal.pone.0234380

**Published:** 2020-06-15

**Authors:** Elie De Lestrange-Anginieur, Chea-su Kee

**Affiliations:** 1 School of Optometry, Hong Kong Polytechnic University, Hong Kong SAR, China; 2 Interdisciplinary Division of Biomedical Engineering, Hong Kong Polytechnic University, Hong Kong SAR, China; Nicolaus Copernicus University, POLAND

## Abstract

It is well-known that correction of blur can improve visual perception. However, it is unclear how the beneficial effect of correction is affected by the regions of correction and the spatial uncertainty introduced by the retinal stimulation. The purpose of this study was two-fold: first, to compare the impacts of blur correction between isoeccentric locations of the visual field; and second, to evaluate the effect of spatial cueing in each corrected location on performing a simple task. Five subjects were asked to complete a simple detection task of a small dark spot stimulus presented randomly at four cardinal retinal locations (eccentricity: 5°) under manipulation of attention via an exogenous cue. Both clear and blurred targets were randomly displayed across the visual field and viewed monocularly through a vision simulator, used to minimize peripheral ocular aberrations. Results confirmed the advantage of clear vs/ blurred images under spatial uncertainty. It was also found that the visual benefit from blur correction is unequal at isoeccentric locations, even for a simple detection task. While manipulation of attention in the presence of spatial uncertainty significantly modulated response time (RT) performance, no differential effect was observed for clear and blurred stimuli, suggesting that attention has only a small effect on the optical benefit for a simple detection task when the display is depleted (no distractor). Those observations highlight the importance of field performance asymmetries in optical interventions and may offer useful implications for the design of extrafoveal refractive correction. Further studies are needed to elucidate how the focus of attention interacts with the perceived gain of vision correction.

## Introduction

Due to the intrinsic presence of blurred images in the retina, any optical ocular correction constitutes an appropriate compromise, involving prioritization of blurs at a given location and/or over the extent of the retina. The prioritization of a clear foveal image over peripheral blurred images best illustrates this compromise, e.g., in progressive addition lenses [1]. However, the visual rule dictating the selection and weighting of optical targets neighboring the angle of gaze has received little attention and is ill-defined when extending the patch of optical ocular corrections [2]. Conventional optical correction follows a strategy of minimization of blur, founded on the view that localized blurs (at the fovea [3] and in the periphery [4]) limit visual performance. This view has been supported by the strong correlation between visual performance and optical quality [5–8], albeit in contradictory observations [9].

Until the interaction between optical and neural filters is fully elucidated [10], the complete elimination of aberrations remains dubious. It is important to ascertain whether optical filtering predominates over neural filtering, and so is likely to fully determine the target of an optical ocular correction. A striking finding is the existence of blur preferences to habitual optical blurs [11–13] (as compared to unwonted optical blurs, such as rotated native blurs), which points to a neural sensitivity adjustment for fine visual patterns of real life images to which the eye is exposed. Despite efforts to reveal the basis of these preferences [14], the spatiotemporal neuronal process underlying neural bias remains undetermined, thus limiting our understanding of their generalization across the ensemble of our visual function. Assuming an optical origin of the relationship between individual optics and neural bias, it has been proposed that long-term adaptation to degraded viewing of eye optics may constitute the cause of such biases [15]. In other words, the eye becomes insensitive to optical information that insufficiently stimulates the eye in order to conserve neural resources. However, whether neural adjustments involve variations in processing (e.g., the manner in which information is processed at the neuronal level) or simply a change of sensitivity due to the neurons’ sensitivity threshold, or both, remains an enigma.

Although the benefits of a full correction can endure individual neural adjustment to blur [16], it appears that this bias can affect the magnitude of a corrective benefit in highly aberrated eyes [17]. Interestingly, this bias not only exists in the fovea, but also in the periphery [18]. With an increasing amount of aberration the eye has to adapt with larger eccentricities, it is therefore plausible that a distributed optical correction may perform better than a total correction of peripheral blur.

Most of the extant literature investigating the relationship between optical and neural filters or visual performance and blur have overlooked the impact of a variable neuron response (visual filter) by restricting the window of the stimulation to a small visual field [4, 18]. The choice of a small visual field leads to uncertainty reduction and a reduction of the effect of noise due to foreknowledge of the target position [19–21]. One reason for this restrictive choice in previous studies is the technological challenge encountered by visual simulators for controlling aberrations over an extended retinal patch, including foveal and extrafoveal regions. Diminished uncertainty regarding where and when the signal is present may assist observers to augment their knowledge of the signal, thus facilitating optical deblurring of the image [22] and selection of an optimal neural filter. An invariable, optimal neural filter might cause optical quality effects prevailing over neural sensitivity in the measured impact of aberration on visual performance, which explains why total blur correction [16] is unaffected by individual neural bias.

Dominant in real life conditions, spatial uncertainty is important in that it can introduce variation in regions of attendance during visual tasks, and thus alter neuronal responses at a given retinal location (e.g., an optimal set of neural filters) [23]. Such variations of spatial attention [24] are known to affect the processing of stimuli, resulting in performance changes in a broad range of visual tasks [25]. Given that most research dealing with attention modulation has not controlled retinal blurs, the impacts of uncertainty on the processing of clear and blur stimuli remain undetermined. Here, the question that we attempt to answer is whether or not blurred images are more affected by positional uncertainty, as compared to clear images. We hypothesize that processing efficiency is not equal for blurred and clear images. To elucidate this issue, we measured the impact of variation in processing caused by exogenous attentional cueing in a detection task. Manipulation of attention was applied via a multi-location acuity task paradigm. To control for non-homogeneous distribution of retinal blurs, a visual simulator featuring a wide-angle diffraction-limited image was employed, which could minimize the aberration of the subject across the visual field. We show here an unequal gain of correction across cardinal retinal locations in a simple detection task when removing bias from the subject’s own blur, unaffected by the focus of attention.

## Methods

### Experimental design

**Fig 1:**
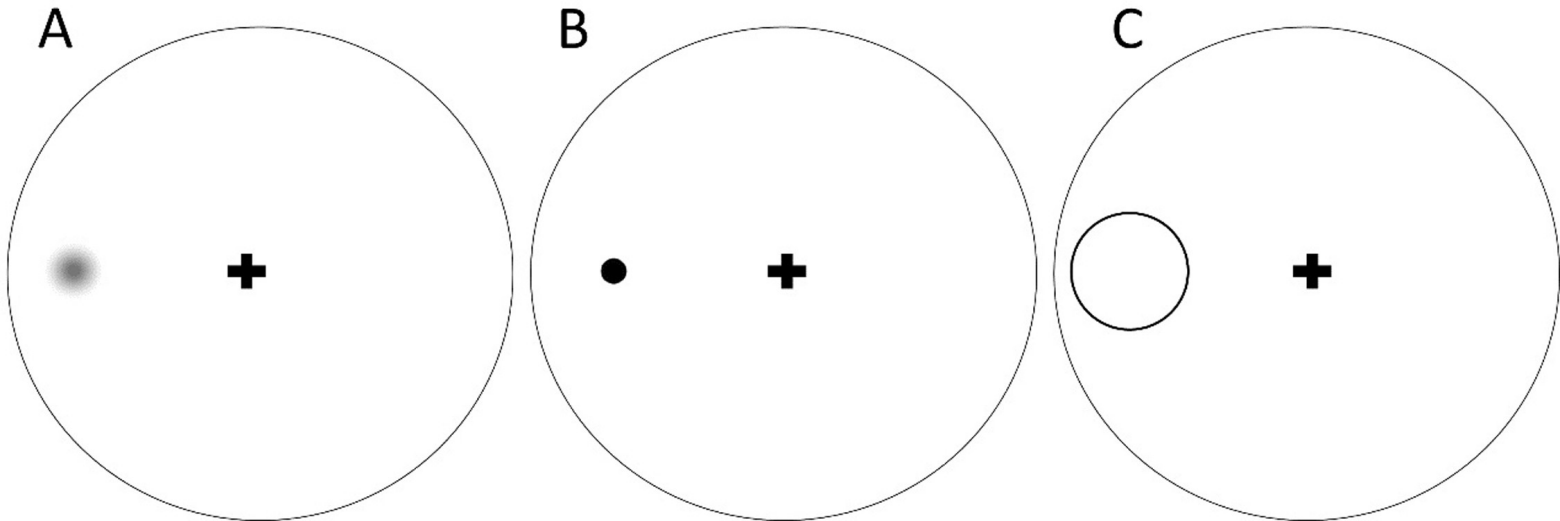
Example stimuli. Schematic diagram showing the fixation cross displayed with (A) a blurred stimulus dot, (B) a clear stimulus dot, and (C) a cue, presented alone against the circular background.

At the beginning of the experiment, subjects were informed that two types of stimuli would appear during the test: a cue and a target stimulus. The observers were asked to fixate on a small cross displayed at the center of the monitor screen (Fig 1, “+”; size, 0.5°x 0.5°) throughout the test.

The subject task was to report the detection of the dark spot signal within the image display. Using a detection task enabled a sufficiently large blur impact in the peripheral retinal locations to compare the variations of blur sensitivity across simulated conditions because blur sensitivity is stronger in detection tasks, as compared to discrimination tasks in the periphery [26–28]. The primary goal of the dot stimulus was to present a broadband, isotropic, and spatially-localized stimulus that would be free from the bias towards specific orientations, features, and regions. This stimulus has been largely used to investigate the processes of spatial attention [29–32]. It was also superior than grating acuity because oriented gratings suffer from meridional neural bias, which may cause orientation-dependent difference in acuity.

All observers were trained with psychophysical procedures, but not informed of the purpose of the experiments. The target stimulus, seen at infinity, consisted of a dark spot briefly displayed (luminance: 0.5 cd/m^2^; exposure time: 33 ms) on a green background (luminance: 15 cd/m^2^; 15° x 15°). A multi-location paradigm was adopted to introduce uncertainty about the location of the target and increase attentional demand. Target stimuli were randomly tested in the near periphery in the four retinal quadrants (i.e., superior, inferior, nasal, and temporal) and centered at ± 5° from the center of the display.

In order to quantify the gain of blur correction, both blurred and clear images were concomitantly tested. Blurred images were computationally generated by convolution of the spot stimulus and a point spread function calculated for a 5 mm diameter pupil [33] and corresponding to a defocus blur set to two waves of RMS wavefront errors (i.e., 1.25D), assuming monochromatic aberrations only with a 550-nm wavelength.

**Fig 2:**
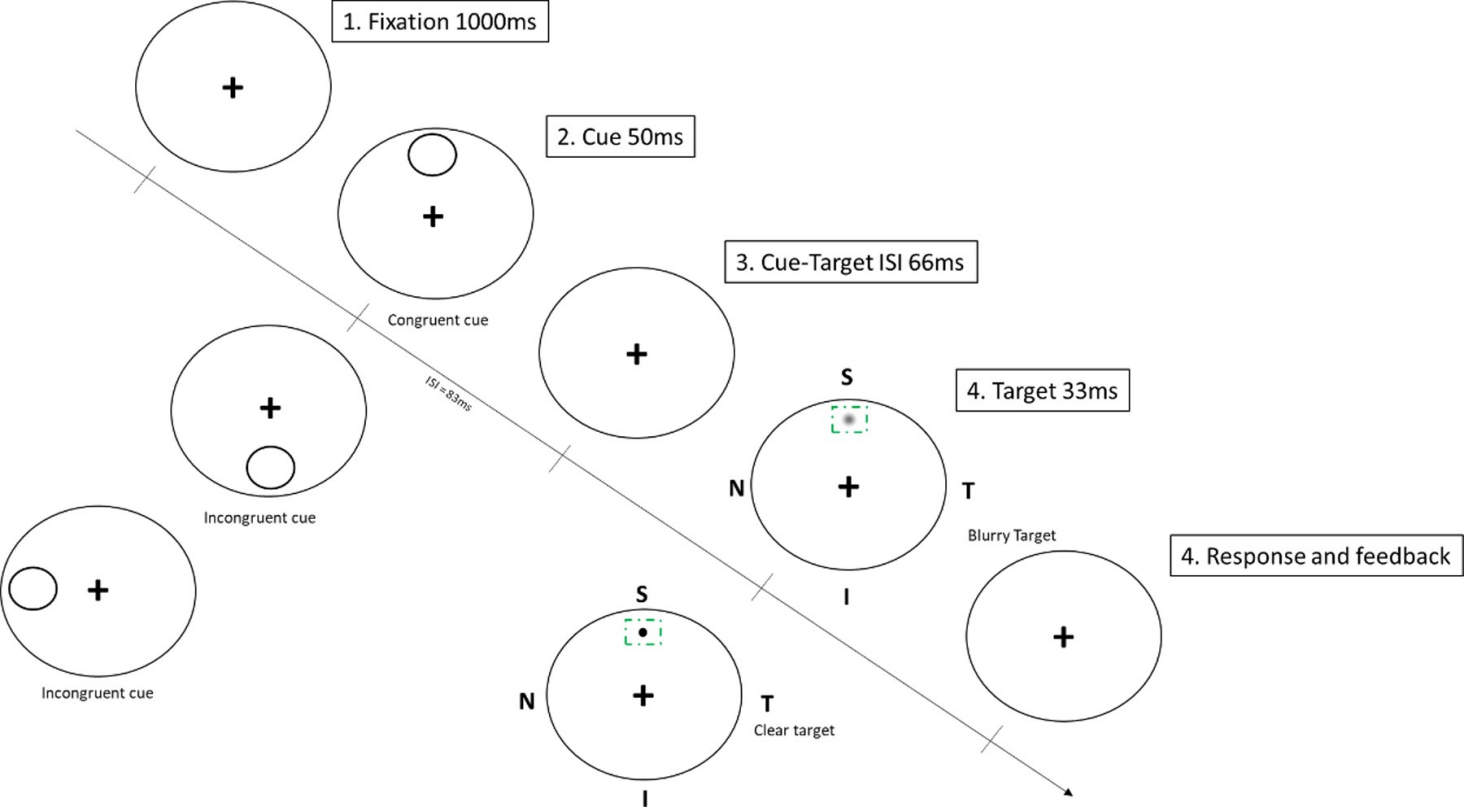
Temporal sequence of the detection acuity task. Detection acuity was tested in the four quadrants of the retina under various combinations of target (clear and blurred) and cueing condition (congruent, incongruent). In the sequence shown above, the observer was required to respond to the dark spot located in the superior retinal quadrant. T, temporal; N, nasal; S, superior; I, inferior. ISI refers to the interstimulus interval.

Manipulation of uncertainty was carried out by introducing sudden and brief changes at a specific location of the display via an exogenous cue (exposure time: 50 ms; interstimulus interval (ISI): 66 ms) displayed immediately prior to onset of the target, as shown in Fig 2.

An exogenous cue was selected to reduce the likelihood of goal- or target-directed eye movements, given that it takes 250 ms for a saccade to occur [34]. The exogenous cue also allowed a rapid test, with less requirement on subjects’ cooperation compared to voluntary attention. The validity of the attentional capture was manipulated by the location of the cue. For the congruent (i.e., valid) condition, the cue center appeared in the same quadrant as the target; in the incongruent (i.e., invalid) condition, the cue appeared in the radially opposite quadrant. Subjects were informed that the cue was uninformative, and the probability of validity was 50%. To minimize interference between the cue and the target, a large ring was used for cueing spatial location. The ring (Figs 1 and 2, duration: 50 ms; diameter: 2.5°; thickness: 1.25 arcmin, black) was centered at the target location (± 5° from fixation) and subtended a visual angle of 2.5° x 2.5° in order to prevent interference between boundaries of the cue and the target. Note that the maximal extent of the visual stimulation was 7.25°.

### Data analysis

An interleaved three-down one-up staircase procedure designed to converge at a detection size producing 79.4% correct responses was used to equate the difficulty of the task between visual conditions and avoid potential ceiling and floor effects in performance. Altogether, there were 16 interleaved staircases for various stimulus conditions i.e., four locations x two cue conditions x two targets). The detection test was repeated a total of five times for each stimulus condition in five sessions (approximately 2,400 response trials in total), and the average value of the threshold measurements was taken as the detection acuity. Auditory feedback was provided after each response. RT was calculated as the time taken for the subject to respond from the offset of the target. The detection threshold is reported as the minimum angle of detection (MAD), which corresponds to the angle subtended by the dot diameter at the eye.

Detection acuity and response time data were processed using a three-way repeated measures analysis of variance (RANOVA) test (retinal location: temporal, inferior, nasal, and superior; visual blur: clear and blurred; spatial cueing: congruent and incongruent), and *post-hoc* pairwise comparisons were performed with the Bonferroni correction.

### Visual instrument

**Fig 3:**
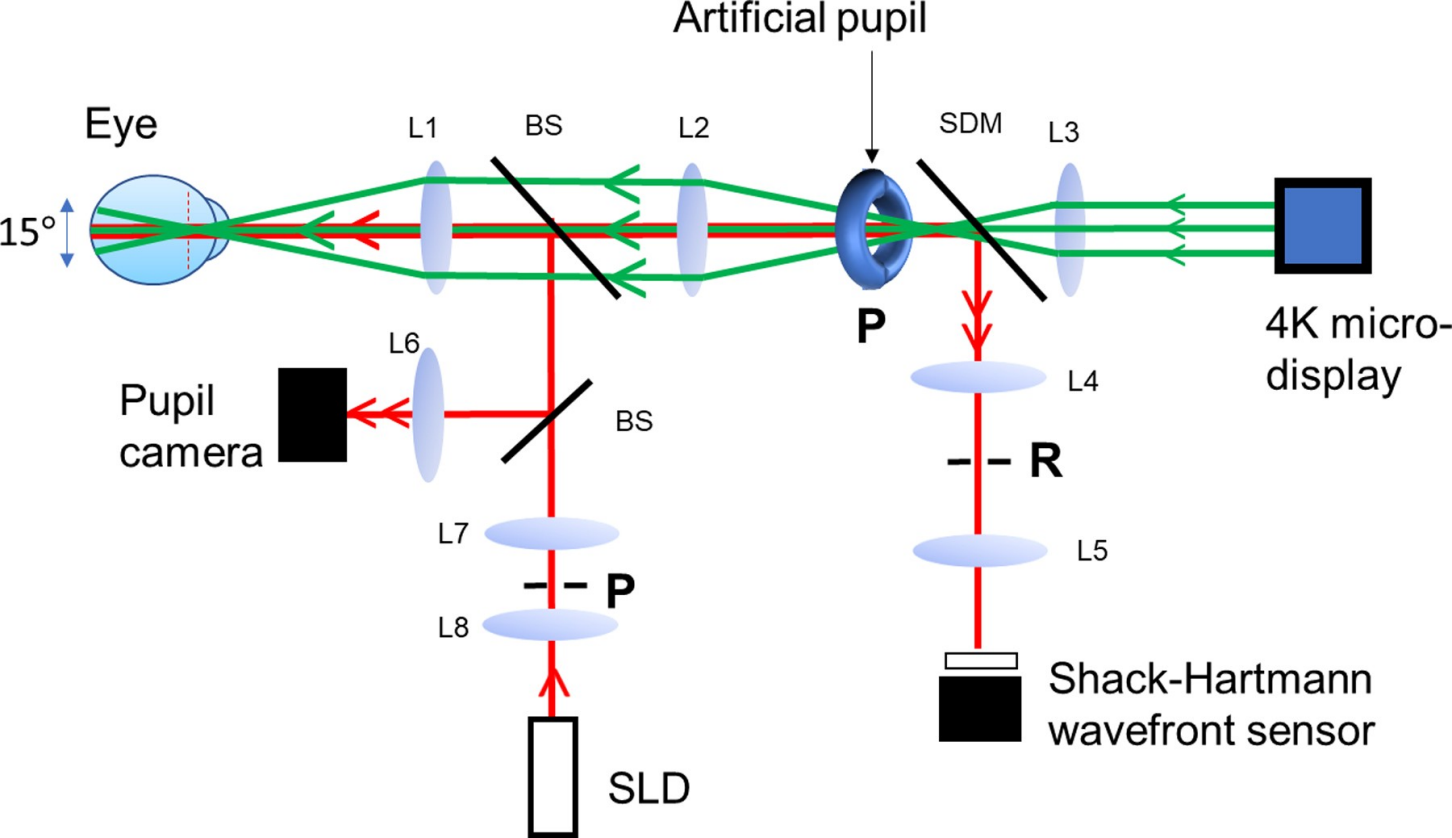
Schematic of the multiscale visual simulator. The green and red lines indicate the chief rays of the optical path. Light exiting the superluminescent diode (SLD) is collimated and sent onto the eye along the illumination path (red), where it forms a small beacon image on the retina. Light is then reflected and sent back along the collection paths (red) for wavefront sensing and pupil monitoring. In the meantime, a wide-angle diffraction-limited image is projected onto the eye along the visual path (green), which is viewed through a small artificial pupil conjugated with the pupil of the eye via a relay lens. L1-L8 lenses, BS, beamsplitter, SDM shortpass dichroic mirror. R and P stands for retinal and pupil conjugate planes.

To display the visual stimulus, a multiscale visual simulator was used that provides a wide-angle diffraction limited image for simultaneously simulating the visual of effect of small and/or large stimuli across the visual field. This instrument was termed a multiscale visual simulator. The purpose of the visual simulator was to ameliorate the measurement comparison between the retinal regions exhibiting differences in blur, while allowing it to simultaneously stimulate multiple locations across the visual field. In brief, the visual simulator is comprised of three branches: an illumination branch, a psychophysical branch, and an analyzing branch (Fig 3).

The illumination branch projects an infrared light beam (*D* = 1 *mm*, *λ* = 840 *nm* ± 50 *nm*) onto the eye using a superluminescent diode coupled to an optical fiber (Superlum, Ireland). Light reflected from the eye is directed to the analyzing branch comprising a wavefront sensor (HASO32, Imagine Eyes, France, 32x40 lenslets) and a pupil alignment control system that are used together to monitor the quality of the retinal images.

The quality of the retinal image is controlled by an adjustable artificial pupil conjugated to the pupil of the eye via a long focal length lens-based telescope (retinal magnification: 0.5). The artificial pupil is designed to provide a wide-angle diffraction limited image, impervious to fixational eye movements, and preset to an optimal diameter of 1.35 mm. This allows minimizing the aberrations of the subject, aberration differences across the field of view, and control aberration variations with pupil changes. Using a three-dimensional translation stage, the position of the subject is adjusted throughout the trials by the experimenter. At the same time, the psychophysical branch presents a highly pixelated image positioned at infinity (DLA-X700R, JVC Inc.) and covering a full visual angle of approximately 27° x 15°. The projection unit consists of three liquid crystal on silicon LCOS microdisplays, which together produce a pixel stimulus approximately the size of a foveal cone (i.e., 0.4 arcmin). Light exiting the projector is filtered via a set of neutral density filters positioned near the pupil plane in order to produce a monochromatic green image (*λ* = 550 *nm* ± 20 *nm*). This highly pixelated image permits simultaneous stimulation of the foveal and extrafoveal retinal regions of the macula, while minimizing light variations susceptible to producing unequal blurring of wide-angle images over space and time. A GeForce GTX980 graphics card driving the projector was used to display the stimuli generated on a PC computer using MatLab software and employed routines from the PsychToolbox [35].

### Experimental procedure

The monocular aberrations of the subjects were minimized using a multiscale vision simulator that projects simulated retinal images, as described below. The RMS wavefront error was approximately the tenth of a wavelength for all the subjects, i.e. only slightly larger than the optical limit imposed by the Marechal criterion for a diffraction-limited system. Additionally, the image quality varies little across visual quadrants, given a mean standard deviation of the RMS wavefront error of about 50nm, across the central (0 degree) and four cardinal (7.5 degrees) locations of the display. This enables bias due to the difference of peripheral blur profile to be ruled out for both the target (5°) and the cue (extending from 3.75° to 7.25°). Since targets were monocularly viewed with a small pupil size and at far distance [36], the fluctuation in accommodation was small as measured by the average variation of Zernike defocus coefficient about 0.25 D of spherical equivalent. This meant that administration of cycloplegic drugs on subject eyes was not required. Data were collected in multiple experimental sessions. Each session lasted approximately 1 h, and each subject completed, an average of five sessions. Every session started with preliminary setup operations that lasted a few minutes. This included a phase of alignment for accurately positioning the observer in the instrument. Subject native defocus was then measured using a Shack-Hartmann wavefront sensor and corrected via a Badal lens by asking the participant to subjectively report the clarity of a Landolt C target of approximately 20 arcmin.

### Subjects

Five subjects (aged 20–38; VA 6/6 or better) with corrected-to-normal vision participated in the experiments. The experimental procedures were approved by the Human Subjects Ethics Committee of the Hong Kong Polytechnic University (HSEARS20170103003), and the research was conducted according to the principles expressed in the Declaration of Helsinki. Written informed consent was obtained from each participant.

## Results

**Fig 4:**
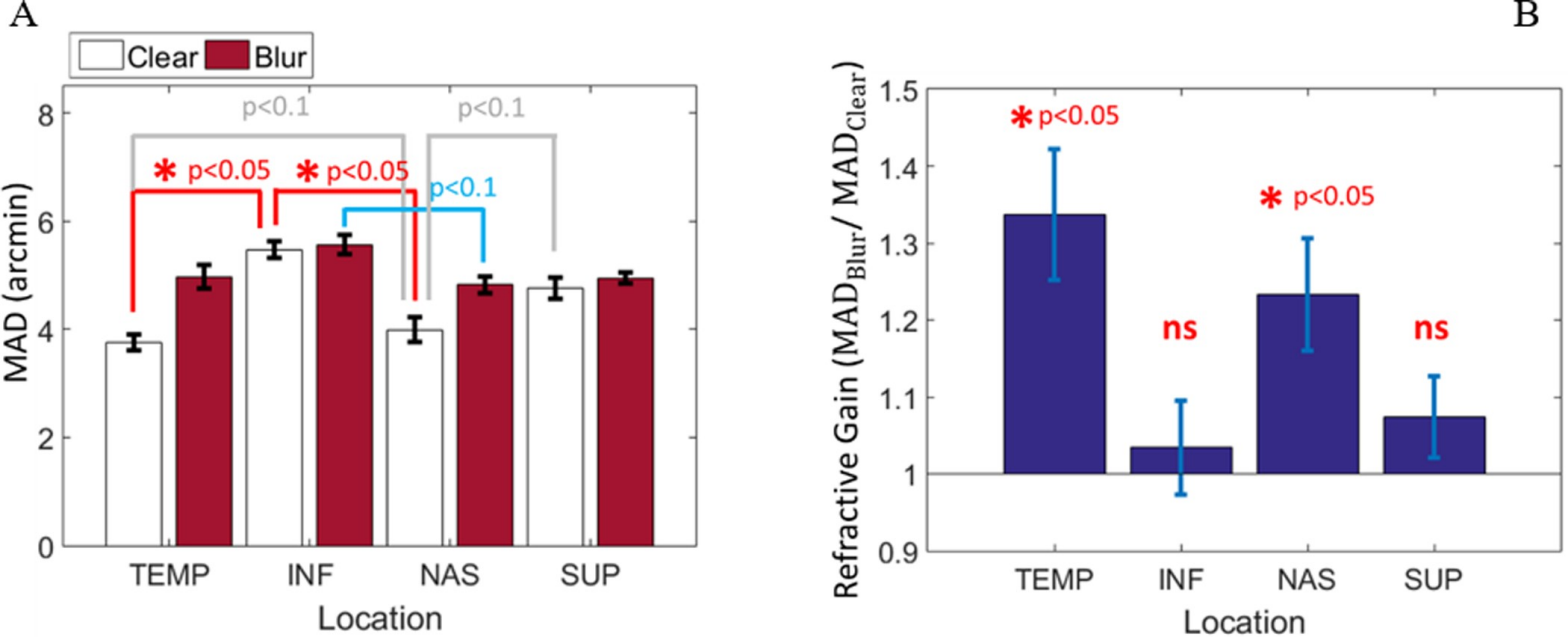
Effect of visual blur on detection in the four retinal quadrants. (**A**) Acuity difference between clear and blurred targets. (**B**) Ratio in MAD between blurred and clear images (*R*_*x*_ = MAD_blur_/MAD_clear_) as a function of retinal location. Blur has only a slight effect on detection acuity in the vertical meridian. Hence, the difference of resolution across foci is unequal at isoeccentric locations of the visual field. In this and following figures, error bars represent one standard error of the mean. Note that ns stands for not significant. TEMP, temporal; NAS, nasal; SUP, superior; INF, inferior.

Spatial location significantly influenced MAD (three-way RANOVA: F(3,12) = 27.367, p<0.001, *η*^2^ = 0.862). As shown in Fig 4A, MAD in the inferior retinal quadrant is significantly poorer as compared to MAD in the horizontal meridian for the nasal (difference MAD: 1.155 ± 0.166 arcmin, p = 0.13) and temporal (difference MAD: 1.113 ± 0.143 arcmin, p = 0.009) retinal quadrants.

Detection acuity is significantly better with clear targets compared to blurred targets (three-way RANOVA: F(1,4) = 16.39, p = 0.015, *η*^2^ = 0.804), showing that processing of blur differences is not altered by the spatial uncertainty of the task. In contrast, clarity has no effect on RT (three-way RANOVA: F(1,4) = 0.129, p = 0.738, *η*^2^ = .031), suggesting that blurring does not influence the temporal processes of the stimulus. Note also that there was no significant three-way interaction between retinal locations, visual blur, and cues for acuity (F(3,12) = 0.739 p = 0.549; *η*_*p*^2^_ = 0.156) and RT (F(3,12) = 2.777 p = 0.087; *η*_*p*^2^_ = 0.410).

The visual benefit of correction is unequal across the visual field, with a significant interaction between location and blurring on MAD (three-way RANOVA: F(3,12) = 4.421, p = 0.026, *η*^2^ = .525). A statistically significant difference in the refractive gain (*R*_*x*_ = *MAD*_*blur*_/*MAD*_*clear*_) was found between locations (Fig 4B, one-way RANOVA: F = 4.648, p = 0.022, *η*^2^ = .537). Along the horizontal meridian, the refractive gain is statistically higher than the refractive gain of 1.0 with mean differences of 0.3371 [(95% CI, 0.1007 to 0.5735), t(4) = 3.96, p = 0.017] and 0.2326 [(95% CI, 0.0312 to 0.4339), t(4) = 3.21, p = 0.033], for the temporal and nasal retinal locations respectively. In contrast, along the vertical meridian, the refractive gain is not significantly different from the refractive gain of 1.0 with differences of 0.0343 [(95% CI, -0.1346 to 0.2031), t(4) = 0.56 p = 0.603] and 0.0743 [(95%CI, -0.0727 to 1.2213), t(4) = 1.40, p = 0.233] for the inferior and superior retinal locations respectively. A paired sampled t-test confirmed a significant difference in the refractive correction gain between the horizontal (*Rx* = 1.2848±0.1756) and vertical (*Rx* = 1.0543±0.1220) meridians; t(9) = 4.58, p = 0.001. A striking observation is that the gain of blur correction in the vertical meridian becomes superfluous for a simple detection acuity task. It is also worthwhile to note that the asymmetry in MAD across the visual field were reduced by the introduction of blur.

**Fig 5:**
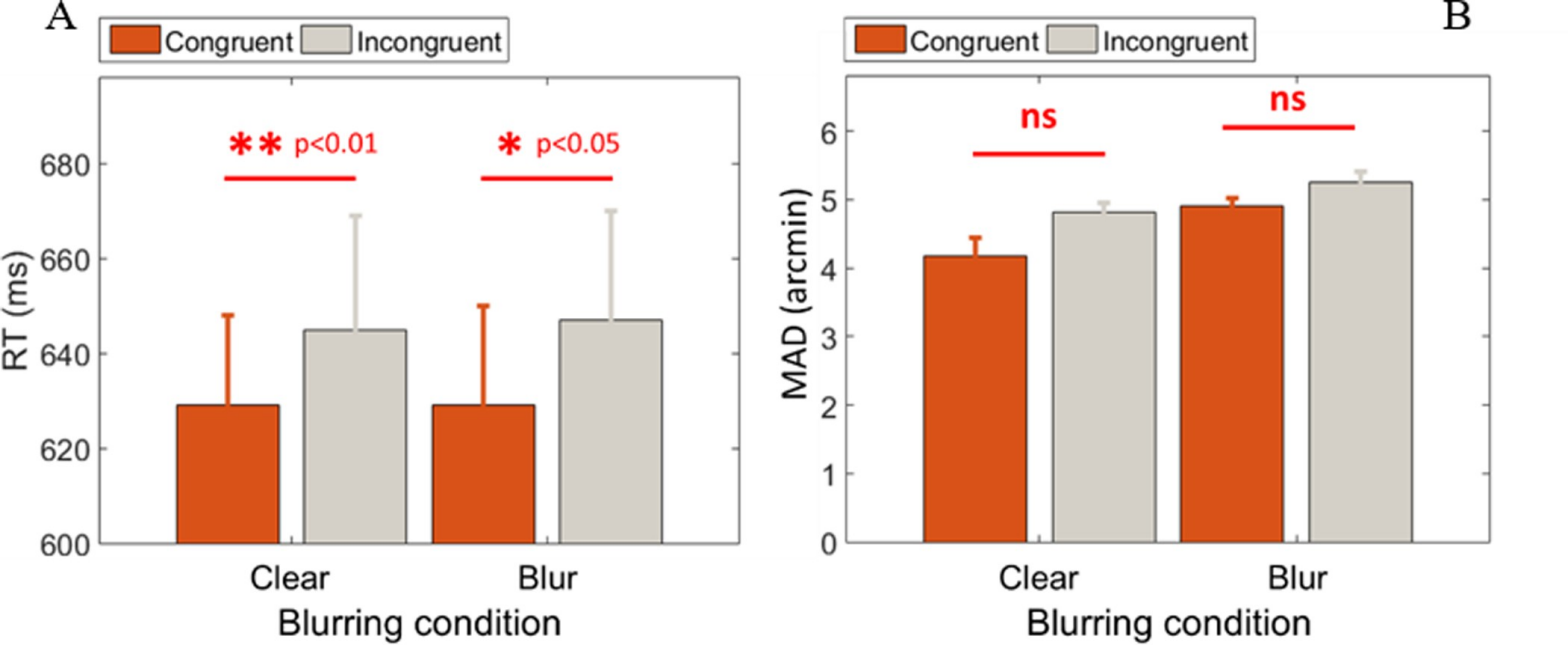
Impact of attention on detection. (**A**) RT and (**B**) MAD as a function of the two cueing conditions for clear and blurred stimulus. There is a significant (p<0.05) and marginal (p<0.1) congruency effect on RT and MAD, respectively. Note that ns stands for not significant.

As shown in Fig 5A, congruency affected performance by enhancing processing speed for both clear and blurred stimuli (three-way RANOVA: F(1,4) = 16.286, p = 0.016, *η*^2^ = .803), confirming that the ring was effective in cueing subjects’ responses. In contrast to RT, congruency showed only a marginal effect on acuity (Fig 5B, three-way RANOVA: F(1,4) = 4.734, p = 0.096, *η*^2^ = .542) for both clear and blurred stimuli, indicating that the processes associated with acuity performance are little affected by manipulation of uncertainty using attentional cues.

## Discussion

Consistent with the existence of anatomical asymmetry [37], and previous findings on field performance, it has been demonstrated that the distribution of visual performance for a simple detection acuity task is unequal at isoeccentric locations across retinal quadrants when the observer’s blur is minimized, with reduced detection acuity in the inferior retinal quadrant as compared to the quadrants of the horizontal meridian. The results also demonstrate that the asymmetry of detection acuity affects the homogeneity of the visual benefit achievable over the visual field (Fig 4B). As anticipated from extant literature [38–41], the beneficial effect of refraction for simple detection acuity tasks exhibit the largest sensitivity in the horizontal meridian. This raises the question of whether or not a uniformly distributed correction (i.e., monofocal correction) is optimal when extending the windows of an optical ocular correction towards more peripheral retinal regions. In order to relax the constraint of optical performance, it could be interesting to include variation of sensitivity caused by neural asymmetry over the field of view in the design of a distributed correction. Nevertheless, the systematic benefit from deblurring images in the detection of blur accords well with the principle of minimizing peripheral ocular aberrations [4]. This result was surprising because it was expected that, under positional uncertainty, the larger image due to the defocused point-spread function could counterbalance its reduced contrast in the detection acuity task.

The difference of acuity between blur and clear images appears little affected by orientation of attention over the visual field in the detection acuity task. This indicates that the exogenous focus of attention mediates stimulus signals, irrespective of the degree of blurring. If so, in a three-dimensional environment, it is possible that the automatic orientation of attention acts independently of stimulus distance. In fact, this result seems to oppose the idea that low contrast stimuli are more affected under spatial uncertainty than suprathreshold, high contrast stimuli [42]. An alternative explanation is that the low level of uncertainty caused by the depleted display (along with the sustained state of attention set by the repetitive pattern of the task) led to a condition in which the existing neural resources were sufficient to optimally process the information modulated by blurring. Further studies are needed to elucidate whether attentional focus is tuned to optical focus. Nevertheless, it appears that blur correction can vary across the visual field to which the subject attends, regardless of the orientation of transient attention. These results may offer useful implications for optical ocular correction aimed at correcting spatially-varying blur of the human eye.

## Conclusions

In summary, the application of a wide field visual simulator for the evaluation of blur sensitivity under conditions of spatial uncertainty introduced by an extended display was demonstrated. When minimizing the bias from the eye own’s blur, it was found that the beneficial effect of refraction in a simple detection acuity task is stronger in the horizontal meridian, and little affected by attentional cueing in a depleted display. This suggests that, when correcting peripheral ocular blurs, special attention should be paid to the visual field inhomogeneities.

## Supporting information

S1 Table On- and off-axis aberrations. Root-mean-square wavefront error (RMSWFE) measured on (0°) and off-axis (7.5°) over a 1.35mm diameter artificial pupil for each subject.
(XLSX)

S2 Table Acuity detection thresholds. RT and MAD values as a function of location, clarity and cueing for each subject.
(XLSX)
